# Development of Polymeric Films Based on Sunflower Seed Proteins and Locust Bean Gum

**DOI:** 10.3390/polym16131905

**Published:** 2024-07-03

**Authors:** Layla Talita de Oliveira Alves, Pãmella Fronza, Idalina Gonçalves, Washington Azevêdo da Silva, Leandro S. Oliveira, Adriana S. Franca

**Affiliations:** 1 Programa de Pós-Graduação em Ciência de Alimentos, Universidade Federal de Minas Gerais, Av. Antônio Carlos, 6627, Belo Horizonte 31270-901, MG, Brazil; laylatalitaoalves@gmail.com (L.T.d.O.A.); pamellafronza@hotmail.com (P.F.); leandro@demec.ufmg.br (L.S.O.); 2 CICECO—Aveiro Institute of Materials, Department of Materials and Ceramic Engineering, University of Aveiro, 3810-193 Aveiro, Portugal; idalina@ua.pt; 3 Departamento de Engenharia de Alimentos, Universidade Federal de São João Del-Rei, Rodovia MG 424, km 47, Campus Sete Lagoas, Sete Lagoas 35701-970, MG, Brazil; was@ufsj.edu.br; 4 Departamento de Engenharia Mecânica, Universidade Federal de Minas Gerais, Av. Antônio Carlos, 6627, Belo Horizonte 31270-901, MG, Brazil

**Keywords:** bioplastics, by-products, galactomannans, protein

## Abstract

Most polymeric food packaging materials are non-biodegradable and derived from petroleum, thus recent studies have focused on evaluating alternative biodegradable materials from renewable sources, with polysaccharides and proteins as the main types of employed biopolymers. Therefore, this study aimed to develop biopolymeric films based on sunflower proteins and galactomannans from locust bean gum. The influence of the galactomannan amount (0.10%, 0.30%, 0.50%, and 0.75% *w*/*v*) on the physicochemical, thermal, and mechanical properties of cast sunflower protein-based films was studied. Sunflower proteins gave rise to yellowish, shining, and translucid films. With the incorporation of locust bean gum-derived galactomannans, the films became more brown and opaque, although they still maintained some translucency. Galactomannans significantly changed the proteins’ secondary structures, giving rise to films with increased tensile resistance and stretchability. Nevertheless, the increase in the galactomannan amount did not have a significant effect on the film’s thermal stability. The protein/galactomannan-based films showed values of water vapor and oxygen permeability that were slightly higher than those of the pristine materials. Overall, blending locust bean gum galactomannans with sunflower proteins was revealed to be a promising strategy to develop naturally colored and translucid films with enhanced mechanical resistance while maintaining flexibility, fitting the desired properties for biodegradable food packaging materials.

## 1. Introduction

The utilization of non-biodegradable and chemical-based polymers had caused serious concerns due to their undesirable longevity as environmental pollutants. The need for more effective alternatives for waste disposal and pollution control has led to increased interest in the development of natural biopolymer-based materials [1]. Such biopolymers are biocompatible and biodegradable, and are thus rather useful for a plethora of applications in the food industry, including edible films, emulsions, and packaging materials.

Proteins and polysaccharides are the main types of biopolymers used in the production of films for biodegradable packaging [2,3,4]. Proteins can be extracted from food and agricultural processing by-products, such as soybean and sunflower oil press-cakes, and further used to produce biopolymeric films. Protein-based polymeric films are flexible and provide good barriers to oxygen transfer [3]. Furthermore, proteins have a unique capacity to form networks, thus improving the plasticity and elasticity of biopolymer-based materials [5]. Previous studies on protein-based polymeric films have shown that both soy and sunflower protein isolates present good film-forming properties, and the resulting films are characterized by significant smoothness and flexibility [6,7].

The secondary, tertiary, and quaternary structures of proteins facilitate interactions and binding that vary in position, type (e.g., hydrogen, ionic, hydrophobic, and covalent bonding), and energy, leading to improvements in film resistance and flexibility. However, the water permeability is high due to the hydrophilic nature of the molecules. The tightly packed network structure of polysaccharides, on the other hand, leads to the production of films that act as barriers for carbon dioxide and oxygen, but they also have high permeability to water vapor [8]. Therefore, recent studies have aimed to enhance desirable film properties by employing blends of proteins and polysaccharides [9]. The main challenge is to be able to choose synergistic composite ingredients that will provide the desired film properties, along with the use of plasticizers and/or gelling agents [8].

The film-forming properties of several polysaccharides (e.g., starch, chitosan, and galactomannans) have been extensively studied [10,11,12], including locust bean gum (LBG) [13]. LBG, a natural polysaccharide extracted from the fruit of the carob tree, is a galactomannan composed of a β-(1–4)-d-mannan backbone with single α-(1–6)-d-galactose substitutions in a 1:4 ratio of galactose to mannose. Given that LBG is non-ionic, its viscosity and solubility are not influenced by the medium pH. Furthermore, the LBG galactose side chains have a strong synergistic interaction with other polymers, which leads to better stabilization and emulsification, and improvements in both the processing and mechanical properties of polymeric films [14].

LGB has been shown to have synergistic behavior with protein-based biopolymers [15]. Recently, soy protein (SP)-based natural hydrogels were developed with the inclusion of LGB [16]. The combination of protein and carbohydrate in the developed polymers enhanced both the functional and mechanical properties in comparison to SP alone, confirming the synergistic behavior of LGB and soy protein. Furthermore, LGB milling-derived dust (LBDM, a residue containing 56% protein) was used in combination with potato starch to produce bioplastic films [17]. Blending the protein-rich residue with starch led to films that were more rigid and more resistant to water in comparison to the original LBDM films [18].

Although there are several studies combining proteins and polysaccharides to obtain polymeric films, [15,17,19,20,21], none have attempted to blend sunflower protein with polysaccharides. The production of sunflower oil (and consequently of its protein as a residue) is significant in a diversity of areas around the globe, and it is usually widespread in areas where soybean is not produced or is sparsely produced [22]. Furthermore, the interactions of storage proteins with non-ionic polysaccharides with high degrees of substitution have been scarcely explored in the literature.

In this work, it was hypothesized that LBG galactomannans can improve both the barrier and mechanical properties of protein-based films. Therefore, sunflower proteins were used as the main biopolymeric matrix and the influence of adding LBG galactomannans (at 0.10%, 0.30%, 0.50%, and 0.75% *w*/*v*) on the physicochemical, thermal, and mechanical performance of sunflower protein-based films was investigated.

## 2. Materials and Methods

### 2.1. Materials

Sunflower seed proteins (SSPs) with low contents of chlorogenic acids (SUNPROTEIN, ca. 80% crude protein) were supplied by “Bio Technologies” LLC (Moscow, Russia) and locust bean gum (LBG, molecular weight ca. 1000 kDa) was purchased from Sigma-Aldrich Co. (St. Louis, MO, USA).

### 2.2. Film Preparation

Films were prepared by solution casting [13,23]. Galactomannan and protein solutions were prepared by dissolving the respective powders in distilled water (LGB: 1% *w*/*v*, 1 h stirring at 25 °C followed by 30 min stirring at 80 °C; SSP: 2% *w*/*v*, 30 min stirring at 25 °C and pH adjusted to 12 with 2M NaOH). For the blended films, the solutions were mixed in the following proportions: 2% SSP (base) + LBG added at 0.1, 0.3, 0.5, and 0.75% *w*/*v*. For all the prepared films, glycerol (SSP: 0.6% *w*/*v*; blended films: 2.0% *w*/*v*) was added as a plasticizer. The determination of the required amount of glycerol was based on preliminary tests, in which film cohesiveness, elasticity, and resistance were observed. The blended films required a larger amount of glycerol in comparison to the pure protein one to improve the interaction between protein and carbohydrate chains. Nonetheless, further increases in the amount of glycerol used in the pure protein solution resulted in sticky films, so it was not possible to add the same amount of glycerol used for the blends. The film solutions were placed in silicone trays and kept at 30 °C for 24 h. All the films were conditioned at 25 °C and 50% relative humidity (RH) for 48 h.

It should be pointed out that, in the case of the protein filmogenic solution, a high pH value (12) was employed. The optimum pH for protein solubilization and subsequent film formation varies with the type of protein and its surface characteristics strictly related to the concentration and arrangement of hydrophilic and hydrophobic amino acid functional groups [24]. There are two main types of proteins in sunflower seeds: water-soluble albumins (sunflower albumins, 2S) and salt-soluble globulins (helianthinin, 11S). Helianthinin is scarcely soluble in water. Previous studies dealing with these proteins reported optimum solubilities at pHs of 10 to 12 [24,25,26,27]. At pHs ranging from 6.3 to 9.7, helianthinin is present in solution in a hexameric form, whereas in the range of 10 to11, it dissociates into a trimeric form. At pH 12, complete and partial dissociation into monomeric and oligomeric forms (2S–3S and 7S, respectively) occurs [24,26], rendering it more soluble.

A high pH value could be of concern in terms of protein denaturation as it is detrimental to the solubility of the protein, since the unfolding of the molecule exposes the less polar and nonpolar groups that were keeping the protein folded together. However, Molina et al. [26] determined that, at pH 12, the amount of denatured sunflower protein was not significant, with approximately 20% of the helianthinin being denatured with respect to the maximum value observed at pH 7.

### 2.3. Film Characterization

#### 2.3.1. Digital Images, Chromatic and Microscopic Properties

A tristimulus colorimeter (ColorFlex, Hunter Associates Laboratory, Reston, VA, USA) with standard D65 illumination and a normal colorimetric observer angle of 10° was employed for the measurement of L* (luminosity), a*, and b*. The values of a* and b* were then employed to evaluate the parameters c* (chroma), which can be directly associated with color intensity, and *h* (hue angle), representing color tone:

(1) $$c^{*}={\left[{\left(a^{*}\right)}^{2}+{\left(b^{*}\right)}^{2}\right]}^{1/2}$$

(2) $$h={tan}^{-1}\;\left(b^{*}/a^{*}\right)$$

Opacity was evaluated as follows [14]: a piece of film (4 × 1 cm) was applied to the inner wall of a quartz cuvette, and the absorbance value at 600 nm was read using a UV spectrophotometer (Biosystem, Curitiba, Brazil). Each measurement was performed in triplicate, and film opacity was calculated as

(3) $$Opacity=A_{600}/X$$

where *A*_600_ corresponds to the absorbance at 600 nm and X represents the film thickness. An empty cuvette was used as the reference opacity.

The film surface was observed using a tabletop scanning electron microscope (model TM4000PlusII, Hitachi, Tokyo, Japan). Each film sample (ca. 1 cm^2^) was placed in the sample holder with carbon double-sided adhesive tape. No overcoating was required and a low vacuum mode was used. The images were obtained at 200-, 500-, and 1000-fold magnifications by applying an acceleration voltage of 5 kV.

#### 2.3.2. FTIR Spectroscopy

The Fourier Transform Infrared (FTIR) spectra of the samples were recorded using a Shimadzu IRAffinity-1 FT-IR Spectrophotometer (Shimadzu, Tokyo, Japan) with a DLATGS (Deuterated Triglycine Sulfate Doped with L-Alanine) detector and an Attenuated Total Reflectance (ATR) accessory with a ZnSe crystal. The scan range was set at 4000–600 cm^−1^, with 45 scans per sample at a 4 cm^−1^ resolution. Measurements were performed in a controlled environment at 20 °C and 50% relative humidity.

#### 2.3.3. X-Ray Diffraction (XRD)

An X-ray diffractometer (D2 Phaser, Bruker AXS GmbH, Karlsruhe, Germany) using CuKα radiation (1.542 Å) and operating at 30 kV was employed. Data were recorded in the range (2θ) of 5–80° with a step size of 0.6°.

#### 2.3.4. Thermal Profile

Thermal profiles of the films were studied and compared using thermogravimetric analysis (TGA). TGA was conducted using a TA Instruments Equipment (model SDT Q600, New Castle, DE, USA). Approximately 10 mg of the samples was placed in an aluminum pan and measurements were carried out between 25 and 600 °C with the temperature increasing at a rate of 10 °C min^−1^ under a nitrogen atmosphere of 100 mL min^−1^ [28].

#### 2.3.5. Thickness and Mechanical Properties

Film thickness was determined using a digital micrometer with 0.001 mm accuracy (Mitutoyo Corporation, Kanagawa, Japan). The measurements were performed at 10 random points for each sample, with three repetitions for each treatment. Mechanical properties, including tensile strength (TS), elongation at break (EB), and elastic modulus (EM), were measured using a texture analyzer (TAXT2plus, Stable Micro Systems, Godalming, UK) and a 5 kg load cell with a deformation speed of 1 mm per min [29]. The samples used for measurements were 25 × 100 mm, and at least 6 replicates were performed for each film.

#### 2.3.6. Moisture Content (MC), Water Vapor and Oxygen Permeabilities (WVP, OXP), and Water Solubility (WS)

The conventional gravimetric method [30] was employed to determine the moisture content (MC) of the samples, with 2 × 2 cm film samples being dried at 105 °C for 24 h.

Water vapor permeability (WVP) was determined using the method described by do Lago and co-workers [31]. Film samples were placed at the opening of glass vials containing previously dried silica, maintained in a controlled environment (20 ± 0.5 °C at 75% relative humidity), and weighed every 24 h for a period of seven days. A calibration curve was built for the evaluation of the silica weight gain over time. WVP (g mm/m^2^ h kPa) was then calculated according to Equation (4):

(4) $$WVP=\frac{w}{t}\times \frac{\delta }{∆P\times A}$$

where *w*/*t* is the slope of the weight gain line as a function of time (g/day); *δ* is the mean sample thickness (mm); *A* is the sample permeation area (m^2^); and Δ*P* is the vapor saturation pressure at the test temperature (2.33921 kPa). Five repetitions were performed for each sample.

Oxygen permeability (OXP) evaluation was performed according to the procedure described by Cheng and collaborators [14]. The prepared film was used to cover the top of a weighted 15 mL flask tube containing 3 g of the deoxidizer reactant (iron powder, activated carbon, and sodium chloride). The flask was then placed in a desiccator containing a saturated solution of barium chloride (90% RH) for 48 h at 25 °C. The original and final weights were recorded, and *OXP* was calculated as

(5) $$OXP=\left(Wf-Wi\right)/\left(t\times A\right)$$

where *Wf* and *Wi* correspond to the final and initial weights (g), respectively, *t* is the equilibration time (h), and *A* is the film area (m^2^). Each measurement was performed in triplicate.

Water solubility (*WS*) was determined according to the procedure described in [32]. The films were cut into 2 cm diameter circles and dried in an oven for 24 h at 105 °C to determine the initial dry weight (*Wi*). They were then submerged in 50 mL of distilled water and homogenized in orbital shaker at 150 rpm for 24 h. Samples were dried and weighed again (*Wf*). *WS* was then calculated using the following equation:

(6) WS=(Wi−Wf)/Wi×100%

#### 2.3.7. Film Biodegradation

Biodegradability assays were performed to assess the potential of the prepared films as an alternative to synthetic ones that usually require rather long times for degradation [33]. The biodegradability test was performed according to the methodology described by Fronza et al. [33]. The soil preparation was based on ASTM G160-03 [34]. Plastic containers (12.5 cm height) were filled with soil up to a height of 4 cm. The prepared film samples (2 × 2 cm) were buried in the soil and the containers were kept at room temperature. Soil moisture was maintained by regularly spraying water and film samples were removed and analyzed daily until complete degradation (8 days).

#### 2.3.8. Statistical Analysis

All experiments were performed in triplicate, with at least 3 measurements per sample. Analysis of variance (ANOVA) was performed using the Minitab 16 software and significant differences (*p* < 0.05) between the treatments were evaluated using Tukey analysis.

## 3. Results

### 3.1. Digital Images, Chromatic and Microscopic Properties

**Figure 1:**
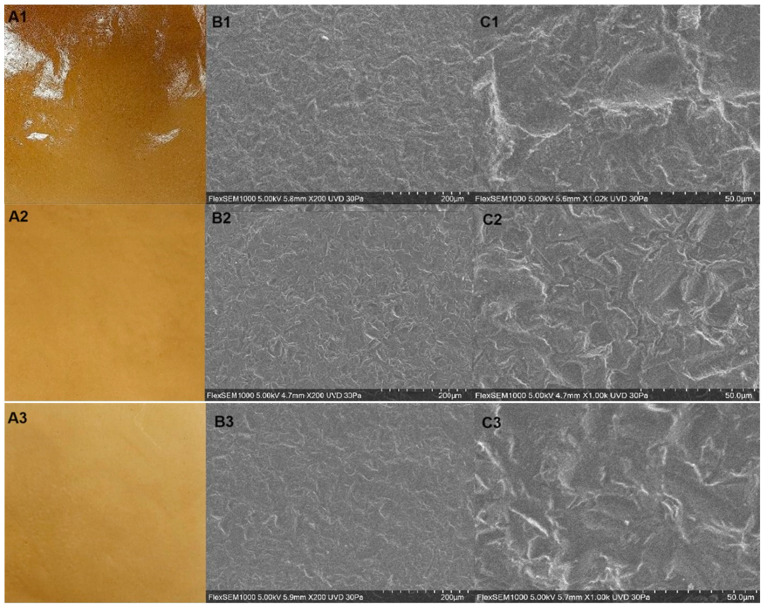

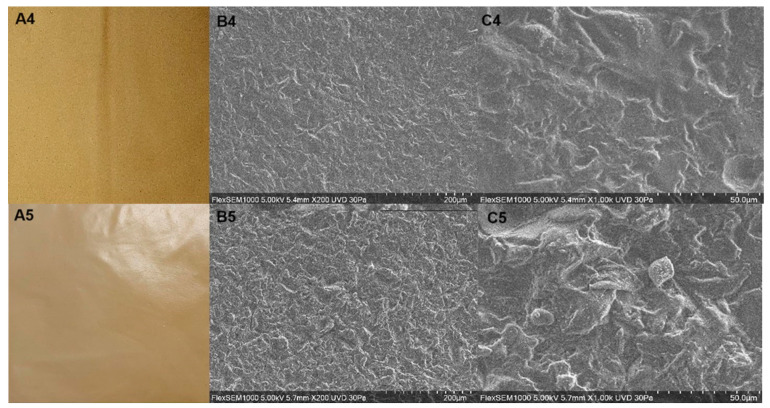
Film photography (**A**) and SEM images (**B** ×200, **C** ×1000)—(**1**): 2% SSP; (**2**): 2% SSP + 0.1% LBG (**3**): 2% SSP + 0.3% LBG; (**4**): 2% SSP + 0.5% LBG; (**5**): 2% SSP + 0.75% LBG.

Images of the prepared films are displayed in Figure 1(A1–A4), and their color parameters are shown in Table 1. The films looked dark orange/brownish, without visible irregularities. Luminosity values, which can range from 0 (black/dark) to 100 (white/light), were very low for the pure protein film. Although addition of LBG to the films resulted in a slight increase in luminosity, the blended films were still dark (L* < 30). The color tone, measured by the hue angle, tended to be orange (~60–80°) for the pure protein and blended films. Color and opacity are important film parameters because they affect the visibility of the packaged materials. Depending on their capability for transmitting light, packaging materials can be classified as (i) transparent, with most of the incident light being transmitted; (ii) translucent, with light being transmitted but also diffused; or (iii) opaque, when no light is transmitted [35]. The films obtained in this study were found to be translucent. The results in Table 1 show that the addition of LBG resulted in an increase in film opacity. Although low opacity and high luminosity are interesting to allow for product visibility, the films herein prepared still allow for product visualization while providing a barrier to light degradation, thus being of interest for the packaging of whole foods or products with functional claims.

The surface morphology of the prepared films was examined using scanning electron microscopy (SEM), and the obtained images are show in Figure 1(B1–B4,C1–C4). The pure protein film (Figure 1(B1–B4,C1)) as well as the blended ones (Figure 1(B1–B4,C2–C5)) presented similar structures, with a uniform dispersion of the polymers in the film matrix, a few agglomerations, and some rugosity, but without any cracks or pores. These images indicate that the polymers interacted with each other to form a uniform film.

### 3.2. FTIR Spectroscopy

**Figure 2:**
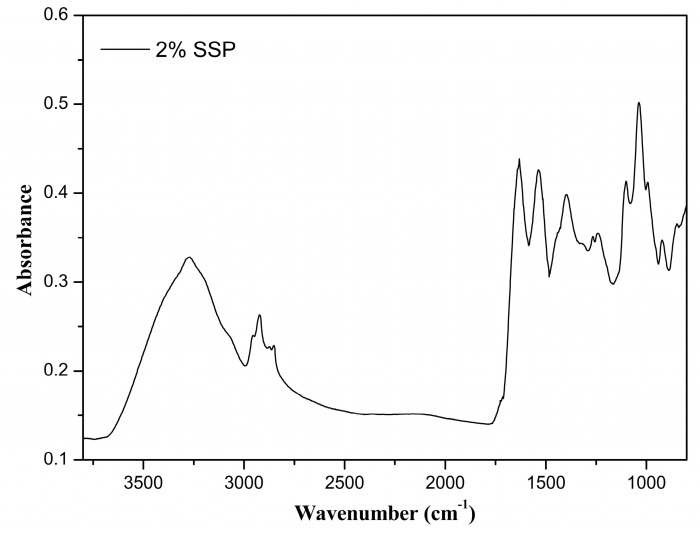
Normalized FTIR spectrum for pure sunflower seed protein film (SSP).

A typical normalized FTIR spectrum for the pure SSP films (SSP) is shown in Figure 2. Bands related to chemical groups that are associated with proteins can be clearly viewed in the SSP spectrum, including a broad band at 3277 cm^−1^ (amide-A), corresponding to O–H stretching, and a band at 2924 cm^−1^ (amide-B), attributed to the asymmetric stretching vibration of COO–H and ${{–NH}_{3}}^{+}$ [36]. Other protein-related bands were also observed at 1631 cm^−1^, attributed to C=O stretching in the amide I bond; 1535 cm^−1^, attributed to a combination of N–H deformation and C–N stretching in the amide II bond (secondary); and 1238 cm^−1^, attributed to a combination of N–H deformation and C–N stretching, comprising the amide III-type of bond [37]. The secondary structural components comprising the amide I region (1700–1500 cm^−1^) can be identified by their respective negative peaks in the second derivative of the FTIR spectrum and the contribution of each component to the overall structure of the protein was estimated using the area under the curve fitted to its related peak in the spectrum [38] (Figure S1, Supplementary Material). The peaks centered at 1631 and 1537 cm^−1^ were attributed to intramolecular β-sheets comprising 69% of the protein secondary structures, whereas the peak centered at 1559 cm^−1^ was attributed to intermolecular parallel β-sheets comprising 1% of the protein secondary structures [36,39]. The peak centered at 1659 cm^−1^ was associated with either α-helix or random coil structures [39] and represents 31% of the secondary structures. Other distinguishable peaks in the second derivative plot were those at 1600 and 1213 cm^−1^, associated with the stretching vibrations of phenyl rings in phenylalanine and tyrosine; one at 1296 cm^−1^, associated with the rocking vibrations of C–H in phenyl groups; and one at 1195 cm^−1^, associated with the in-plane bending vibration of phenyl rings in phenylalanine and tyrosine.

Although at high pH values the protein structure tends to loosen, dissociate, and unfold due to disruption of inter- and intramolecular hydrogen bonds [36], the analysis of the FTIR spectrum and the respective second derivative demonstrated that, during film formation, the proteins were able to retain some of the secondary structures that are characteristic of globular proteins.

Although the FTIR spectrum and the respective second derivative plot allowed for the identification of peaks characteristic of proteins (e.g., amide I-, amide II-, and amide III-type bonds) and of their respective secondary structures (e.g., β-sheets and α-helix structures), they clearly demonstrated that other classes of compounds are also present, such as polyphenols. The sharp band at 1691 cm^−1^ can be attributed to C=O stretching vibration in chlorogenic acids and their derivatives (e.g., quinones), whereas the band at 1296 cm^−1^ can be attributed to the rocking vibration of C–H groups in phenyl rings. The bands at 1180 and 1114 cm^−1^ were, respectively assigned to C–OH groups and to the phenyl ring bending vibrations in chlorogenic acids and their derivatives [40]. The prominent peak at 1600 cm^−1^ was assigned to the stretching vibration of phenyl rings and the peak at 1627 cm^−1^ was attributed to the stretching vibration of the C=C ethylenic group in phenyl rings. In the processes of sunflower seed oil extraction and protein isolation, chlorogenic acids and derivatives are known to react (e.g., oxidation and dimerization) and bind themselves to the seed proteins, forming covalent and non-covalent types of bonds [41]. One type of covalent bond that is formed is imine bonds. The sharp band at 1612 cm^−1^ (Figure 2) can attributed to the stretching vibration of the azomethine CH=N bond and the bands in the range of 1583 to 1570 cm^−1^ can be attributed to the stretching vibration of the phenyl groups associated with imines, thus indicating the possibility of some of the chlorogenic acid derivatives being linked to the proteins by this type of bond.

**Figure 3:**
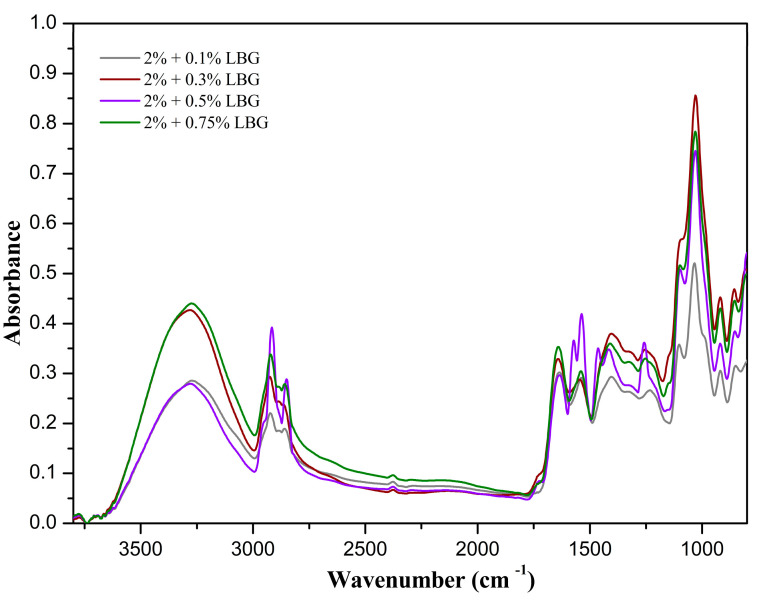
Normalized FTIR spectra for blend films.

The normalized FTIR spectra for the films of the protein–polysaccharide blends are presented in Figure 3. It is rather clear that there were protein–polysaccharide interactions in the amide A and amide B regions of the spectra, with the wavenumber for amide A blue-shifting from 3277 cm^−1^ in the pure protein film spectrum to 3284 cm^−1^ in the spectra for the films comprised of blends of protein and galactomannans. The wavenumber at 2924 cm^−1^ in the spectrum of SSP red-shifted to 2916 cm^−1^ in the spectra of the blends. Both the blue shift and the red shift can be explained by hydrogen bond-type interactions between proteins and galactomannans. The blue-shifted hydrogen bond can be explained by a balance between attractive (electrostatic) and repulsive (steric) forces, in which there is an electrostatic attraction between a charged N–H group of the protein and an O–H group of the polysaccharide balanced by a significant repulsion from the O atom due to the limited distance between the N and O atoms, thus causing the bond of the N–H group to be compressed [42]. The red-shifted hydrogen bonds can also be explained by a balance between attractive and repulsive interactions in which strong orbital interactions cause significant bond elongation, overcoming the bond shortening caused by the steric repulsive forces.

Decreases in the heights of the peaks associated with amide I and amide II (1631 and 1537 cm^−1^) were observed in the spectra for the blends when compared to the spectrum for the pure protein film. These are indicative of the degree of hydrogen bonding between proteins and galactomannans necessary to assure the stability of the blends.

The curve-fitted spectra for the protein–galactomannan blend films are presented in Figures S2–S5 (Supplementary Materials). It is clear that the interactions between the polysaccharide and the protein significantly altered the proportions of the secondary structures of the protein. As the content of polysaccharides was increased in the blend, there was a significant reduction in the amounts of both intra- and intermolecular β-sheets, represented by the proportionally gradual reduction in the contribution of these groups to the overall structure. The proportion of β-sheets was at first increased from 69% for the pure protein film to 76% for the blend with 0.1% galactomannan, which subsequently decreased to 52% for the blends with 0.3 and 0.5% galactomannan, and further decreased to 22% for the 0.75% blend. Conversely, the contribution of α-helix/random coil structures initially decreased from 31% for the pure protein film to 24% for the 0.1% blend, subsequently increasing to 48% for the 0.3 and 0.5% blend, and further increasing to 78% for the 0.75% blend. These results demonstrate that the polysaccharide/protein mass ratio plays a significant role in the natures and intensities of the interactions between these two types of molecules. Hence, it can be inferred that during film preparation, the protein molecules primarily partially unfolded and dissociated from each other in the portions dominated by β-sheets structures and only a fraction of these types of structures were retained due to the facilitated interactions of the unfolded and dissociated portions of these molecules with the galactomannans. Galactomannans are non-ionic polysaccharides and will interact with proteins primarily through their terminal mannose unit at the reducing end of the backbone chain and by hydrogen bonds through their hydroxyl groups.

The 1400 to 750 cm^−1^ range of the FTIR spectrum in Figure 3 is clearly comprised of a cluster of unresolved peaks. This range of wavenumbers encompasses the local symmetry region (1400 to 1200 cm^−1^) and the polysaccharide fingerprint region (1200 to 800 cm^−1^), with the range 900 to 800 cm^−1^, titled the anomeric region, allowing for the discrimination of the α and β configurations of the anomeric carbons [43]. The second derivative of the spectrum showed a suitable resolution of the peaks, allowing for a more detailed analysis of the fingerprint region. The peak at 879 cm^−1^ was attributed to C1–H bending vibration of mannose-containing polysaccharides. The peak at 815 cm^−1^ refers to the ring vibration of mannose-containing polysaccharides. A peak at 848 cm^−1^ together with a sharp peak at 925 cm^−1^ indicates the existence of α-configuration glycosidic bonds in the polysaccharide chain, whereas the peaks surrounding 890 cm^−1^ indicate the existence of β-configuration glycosidic bonds in the polysaccharide chain [43]. Both configurations are present in galactomannan for its molecules consist of chains of β(1–4)-linked D-mannopyranosyl units with single α(1–6)-linked D-galactopyranosyl units as side chains. The peaks surrounding 1004 and 1018–1029 cm^−1^ were associated with the C–O and C–C stretching vibrations of C6–H2–O6 in galactomannans [11] and those surrounding 1145 cm^−1^ were attributed to glycosidic bond vibrations (O–C–O) in mannose-containing hemicellulose. The lack of bands at 1740 and 1245 cm^−1^ can be interpreted as an absence of acetyl groups in the galactomannan used [15]. The aforementioned bands verify the presence of galactomannans in the film. However, in the spectrum, there were several other peaks that could not be associated with galactomannans or protein. This is expected since it is rather difficult to isolate and purify galactomannans and proteins. The peak at 1342 cm^−1^ bears a contribution of the C–H rocking vibration of cellulose rings [43], and the peak at 1161 cm^−1^ can be associated with glycosidic bond vibrations (O–C–O) in cellulose [44]. The peaks surrounding 1070, 1046, and 981 cm^−1^ were attributed to C–O and C–C stretching vibrations in C6–H2–O6 in cellulose and the small peak at 890 cm^−1^ was attributed to the C1–H bending vibration of cellulose [44]. Hence, it can be safely inferred that cellulose was also present in the protein–polysaccharide film.

It must be pointed out that, at high pH values, a certain degree of protein aggregation has been reported by other researchers [26]. However, in the present work, protein aggregation was not observed. Such aggregations would cause visible changes to the films, mostly represented by heterogeneities in the form of scattered tiny darker spots throughout the film surface. Protein aggregation would also be visible in the film micrographs.

### 3.3. X-ray Diffraction

**Figure 4:**
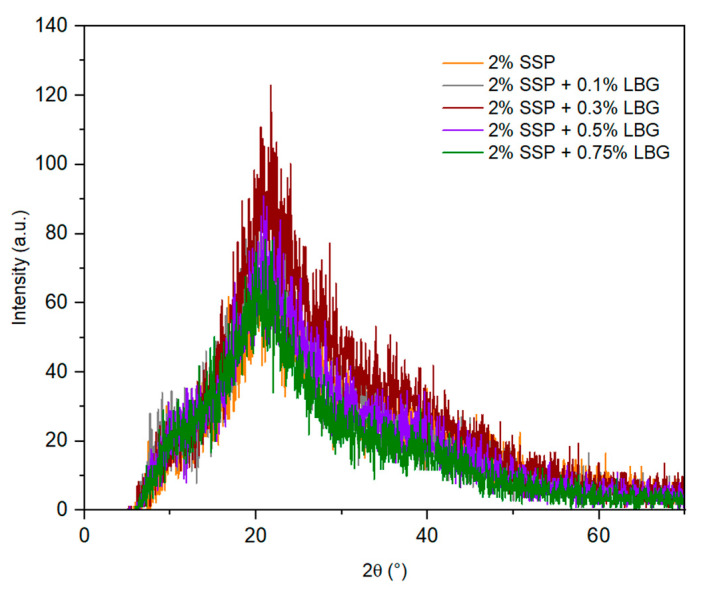
XRD patterns obtained for the prepared films.

The XRD patterns obtained for the prepared films are shown in Figure 4. The pure protein film presented a broad pattern with two broad peaks at 2θ = 9° and 20°. Deng et al. [36] also reported two peaks at 2θ = 10.5° and 20.2° for films prepared with egg white protein, associated with the α-helices and β-sheets in the protein secondary structure. The XRD patterns obtained for the blended films were similar to the one observed for the pure protein film, with variations in peak intensity, corroborating the interactions between the protein and polysaccharides.

### 3.4. Thermal Behavior

**Figure 5:**
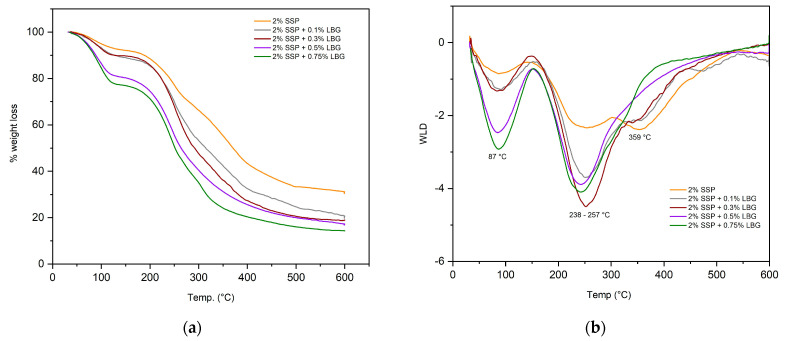
(**a**) Weight loss curves and (**b**) their derivatives for the prepared films.

TGA was employed to investigate the thermal behavior of the prepared films (Figure 5). The TGA curves (Figure 5a) show the variation of weight loss with temperature, with the derivative of weight loss (DWL) curves (Figure 5b) indicating three significant thermal events for all the prepared films. The first event or decomposition stage, regardless of the type of film, can be associated with the evaporation of water, and the corresponding weight losses were in the range of 7 to 12%. Following water desorption, a two-step decomposition curve was observed for all the prepared films.

In the case of the pure protein film, the main decomposition stage occurred in the range from 147 to 452 °C, with two peaks in the DTG curve at 247 and 359 °C. The first peak corresponded to the decomposition of glycerol. The second peak represents either protein decomposition or the decomposition of galactomannans and proteins in the blended films. Notice that the addition of LGB did not have a significant effect on thermal stability. The maximum temperature observed in the third degradation stage was similar for all the prepared films (358–360 °C), and the temperature degradation range was slightly higher for the blended films (451–600 °C) in comparison to the pure protein one (458–580 °C), given the interaction between protein and carbohydrate chains.

### 3.5. Thickness and Mechanical Properties

The film thickness values are displayed in Table 2. The pure protein film presented the highest thickness value, with the film thickness decreasing as the amount of added LGB increased. As the protein molecules are dissolved in water, relaxation of the polymer network with a consequent volume expansion occurs and, upon the removal of water during drying, the film will be thicker (i.e., less dense). With the addition of polysaccharides to the film-forming solution, the interaction of the polysaccharide molecules with those of the proteins alters the conformation of the components, causing a reduction in the degree of relaxation of the protein chain, thus restricting the network volume expansion, which in turn leads to thinner films upon drying when compared to those of pure protein [45].

The mechanical properties of the prepared films are also shown in Table 2. The TS and EM values observed for the pure protein-based film, 3.45 and 18.58 MPa, respectively, are within the range reported in the literature for sunflower seed protein films [7]. However, EB (125.81%) was significantly high in comparison to some values reported in the literature for sunflower seed protein-based films, ranging from 24% to 34% [7,46]. Nonetheless, other studies have reported higher EB values for protein-based films, in the range of 182% to 231% for soy protein and 250% for sunflower protein [25]. In the latter, both the amounts of protein and glycerol were higher than the ones employed in the present study. Such results indicate that the mechanical properties of the protein-based film depend not only on the protein content but also on the extent that the protein unfolds. The unfolding of the globular structure of the sunflower protein increases protein−protein interactions, which have a direct effect on elasticity [25]. Notice that the addition of LGB led to a significant decrease in elasticity modulus, which can be viewed as a measure of the stiffness of an elastic material. This is attributed to the restricted mobility of the protein due to a reduction in its degree of relaxation. Therefore, we observed that, in general, the addition of galactomannans to the protein films led to improvements in the mechanical properties, given that it provided an increase in tensile strength while maintaining film flexibility. A similar behavior was reported for films based on soy protein with added LGB [47].

### 3.6. Moisture Content (MC), Water Solubility (WS), and Water Vapor and Oxygen Permeabilities (WVP, OXP)

The results obtained regarding moisture content, film solubility in water, and film permeability are shown in Table 3. The moisture content of the blends was significantly higher in comparison to the pure protein (control) film. This was due to the difference in the amount of added plasticizer (glycerol), which was much higher for the blended films (2.0% *w*/*v*) in comparison to the pure protein film (0.6% *w*/*v*). The increase in moisture as the LGB concentration increased in the blended films was attributed to the hydrophilic nature of the galactomannans.

The desired water solubility value of a polymeric film depends on its application or intended use. For instance, low solubility may be required to obtain a moisture-resistant package to maintain product integrity. Nonetheless, use of the polymer for the encapsulation of food ingredients requires high solubility [6]. The highest solubility was observed for the pure protein film (see Table 3). The concentration and arrangement of hydrophilic and hydrophobic amino acid groups at the protein surface have a significant effect on its behavior in a solution. Protein solubility increases at pH levels above and below the isoelectric point, due to an increase in electrostatic repulsion induced by positive and negative net charges on the protein surface. Sunflower protein isolates have been reported to present minimal solubility (20–30%) in the pH range of 4.0 to 6.5 and high solubility (80–95%) at pH 10 [24]. Therefore, the use of an alkaline pH in the preparation of the pure protein film had a significant effect on its solubility. LGB, on the other hand, is non-ionic, so its solubility is not affected by pH. The LBG film solution was subjected to thermal treatment at 80 °C to attain solubility. Notice that the solubility of the blended films tended to decrease as the amount of LGB increased. Given that LBG is hydrophilic, its addition should increase solubility. However, a previous study using whey protein and LGB also reported the same behavior observed here, this being attributed to a cross-linking effect caused by the thermal treatment [23].

One of the most common uses of biopolymeric films is to control the transfer of water between the packaged food and the environment [14]. WVP values, shown in Table 3, can provide an estimate of this behavior. Many factors affect WVP, including film thickness, interactions among polymeric chains, the presence of hydrophilic or hydrophobic groups, and others. The pure protein film presented the lowest WVP value, with the highest values being observed for the blended films. This behavior seemed to be directly related to the different glycerol levels of the films. Glycerol is highly hydrophilic, given its hydroxyl groups. The higher amount of glycerol added to the blends may have enhanced water clustering on the polymer and increased the free volume of the film matrix, thus increasing water diffusivity and consequently water vapor permeability [48]. Nonetheless, the WVP values of all the films were still low in comparison to polystyrene (4–10.8 g mm kPa^−1^d^−1^m^−2^), a synthetic packaging material commonly used for the storage of fruits and vegetables [49]. Increasing the amount of LGB in the blends led to a slight decrease in WVP, a good indication of cross-linking between protein and polysaccharide molecules. Crosslinking should cause a decrease in the availability of hydroxyl groups, thus limiting polysaccharide–water interactions through hydrogen bonding [50].

Oxygen permeability (OXP) is also an important film barrier property, given the possibility of food deterioration due to the oxidation of lipids and other food components. OXP behavior is similar to the one observed for WVP, although the differences were not as significant. The slightly larger OXP values of the blended films in comparison to the non-blended one was probably due to the differences in the amount of added plasticizer. Glycerol improves molecular mobility, allowing for better diffusion of oxygen through the polymeric matrix. Comparing the blended films, OXP tended to decrease as the amount of added galactomannan increased. The addition of LGB contributes towards an increase in the number of hydroxyl (polar) groups in the molecular structure, improving the barrier to oxygen (non-polar) molecules [11].

### 3.7. Film Biodegradation

**Figure 6:**
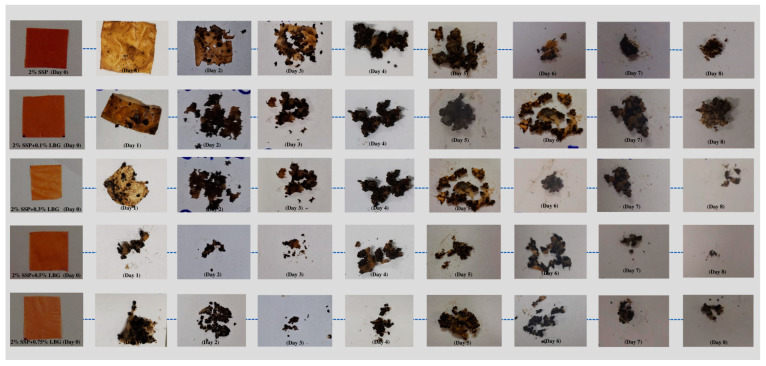
Biodegradability results for the prepared films.

The macroscopic changes that occurred over time as the films remained buried in the prepared soil can be viewed in Figure 6. The mass variation of the buried films over time could not be recorded because soil particles were strongly adhered to the samples. The presence of soil particles resulted in an overestimation of the mass values, while their removal damaged the films, thus resulting in sample losses and consequent underestimation of mass values. Nonetheless, it was possible to observe that all the prepared films degraded entirely after the eighth day of evaluation. Other studies using carbohydrate and protein-based films have reported complete degradation after 4 to 12 days [33,51,52]. Observation of the result variation with time showed that the addition of LBG seemed to favor degradation. A film containing only LGB was prepared for comparison to the other films, and it was observed that degradation was complete after only 3 days (see Figure S6 in the Supplementary Materials). Thus, in the blends, it seems that the LGB degraded first, exposing the protein chains and favoring degradation.

## 4. Conclusions

Biopolymeric films based on distinct blends of sunflower seed proteins and locust bean galactomannans were developed and their respective properties were comparatively evaluated. Sunflower seed protein was used as base and small amounts of galactomannans were added to the film-forming solution. The interactions of the galactomannans and proteins were attested by the changes that occurred in the types of secondary structures of the proteins when the films comprised of blends were compared to that comprised of pure protein (control). The films comprised of blends presented significantly fewer β-sheets and β-turns than the film of pure protein. The addition of galactomannans to the proteins led to more cohesive films with a smaller thickness than that of the film of pure protein and increasing the amounts of galactomannans in the blends led to increasingly higher tensile strengths and decreasingly lower elongations at break, which is consistent with the formation of more cohesive structures promoted by the intensification of interactions between the galactomannans and protein molecules. The types of interactions between galactomannans and protein molecules in the blends, together with the presence of the added glycerol, promoted an increase in the film water vapor permeability in comparison to that of the pure protein film. The water solubility of the blended films was lower than that of the film of pure protein, demonstrating that the polysaccharide molecules really interact with the proteins to form the films. Altogether, these results led to the conclusion that the higher the amount of galactomannan in the blend, the more strong the interactions between the polysaccharide and the protein molecules are. The developed biopolymeric films present potential for applications in the food and other industrial sectors as alternatives to films based on petroleum-derived polymers.

## Figures and Tables

**Table 1:** Film color and opacity.

Treatment	Color Measurements			Opacity (mm^−1^)
	L*	*h*	c*	
2% SSP	23.75 ± 0.05 d	61.23 ± 1.25 c	9.09 ± 0.11 a	6.57 ± 0.05 d
2% SSP + 0.1%LBG	25.68 ± 0.05 c	58.22 ± 2.17 c	6.61 ± 0.19 b	8.86 ± 0.10 c
2% SSP + 0.3%LBG	25.78 ± 0.05 c	72.08 ± 1.29 b	5.81 ± 0.08 c	8.86 ± 0.10 c
2% SSP + 0.5%LBG	27.66 ± 0.11 b	73.69 ± 2.48 b	5.49 ± 0.26 c	9.50 ± 0.17 b
2% SSP + 0.75%LBG	28.63 ± 0.04 a	78.03 ± 2.36 b	4.73 ± 0.29 d	12.11 ± 0.18 a

Means with the same letters in the same column did not show statistical differences (*p* > 0.05).

**Table 2:** Mechanical properties of the prepared films.

Treatment	Thickness (mm)	Tensile Strength (MPa)	Elongation at Break (%)	Elasticity Modulus (MPa)
2% SSP	0.13 ± 0.01 a	3.45 ± 0.40 b	125.81 ± 3.11 a	18.58 ± 0.90 a
2% SSP + 0.1% LBG	0.11 ± 0.01 b	1.89 ± 0.23 c	116.13 ± 6.22 b	4.00 ± 0.28 b
2% SSP + 0.3% LBG	0.09 ± 0.01 c	3.28 ± 0.21 b	113.10 ± 5.44 b	4.37 ± 0.33 b
2% SSP + 0.5% LBG	0.10 ± 0.01 bc	3.47 ± 0.74 b	112.53 ± 2.87 b	4.03 ± 0.59 b
2% SSP + 0.75% LBG	0.09 ± 0.01 c	6.31 ± 0.72 a	131.87 ± 1.39 a	3.60 ± 0.62 b

Means with the same letters in the same column did not show statistical differences (*p* > 0.05).

**Table 3:** Moisture content, solubility in water, and film permeability.

Treatment	MC (%)	WS (%)	WVP (g mm kPa^−1^ d^−1^m^−2^)	OXP (×10^−7^ g mm^−2^s^−1^)
2% SSP	16.20 ± 0.53 f	33.21 ± 0.96 a	0.62 ± 0.01 d	0.99 ± 0.03 bc
2% SSP + 0.1% LBG	42.97 ± 1.17 d	31.43 ± 0.86 a	1.16 ± 0.02 a	1.15 ± 0.04 a
2% SSP + 0.3% LBG	48.10 ± 0.20 c	24.39 ± 0.97 b	1.04 ± 0.02 bc	1.10 ± 0.07 ab
2% SSP + 0.5% LBG	51.40 ± 1.11 b	18.30 ± 0.89 c	1.06 ± 0.02 b	1.02 ± 0.01 bc
2% SSP + 0.75% LBG	56.23 ± 0.15 a	7.18 ± 0.65 d	1.01 ± 0.02 c	0.96 ± 0.01 c

Means with the same letters in the same column did not show statistical differences (*p* > 0.05).

## Data Availability

The original contributions presented in the study are included in the article/supplementary material, further inquiries can be directed to the corresponding author.

## Supplementary Materials

The following supporting information can be downloaded at: https://www.mdpi.com/article/10.3390/polym16131905/s1, Figure S1. Curve fitting of the FTIR spectrum for the pure protein film. Figure S2. Curve fitting of the FTIR spectrum for the blend film with 2% SSP and 0.1% LBG. Figure S3. Curve fitting of the FTIR spectrum for the blend film with 2% SSP and 0.3% LBG. Figure S4. Curve fitting of the FTIR spectrum for the blend film with 2% SSP and 0.5% LBG. Figure S5. Curve fitting of the FTIR spectrum for the blend film with 2% SSP and 0.75% LBG. Figure S6. Biodegradability test results of film based on pure LGB.
