# Supplementary material for: Development of Polymeric Films Based on Sunflower Seed Proteins and Locust Bean Gum
Source: Polymers (Basel). 2024 Jul 3;16(13):1905. doi: 10.3390/polym16131905 (PMC11244352; doi:10.3390/polym16131905)
Supplement: Supplementary file 1 [file polymers-16-01905-s001.zip › polymers-2995276-supplementary.docx]

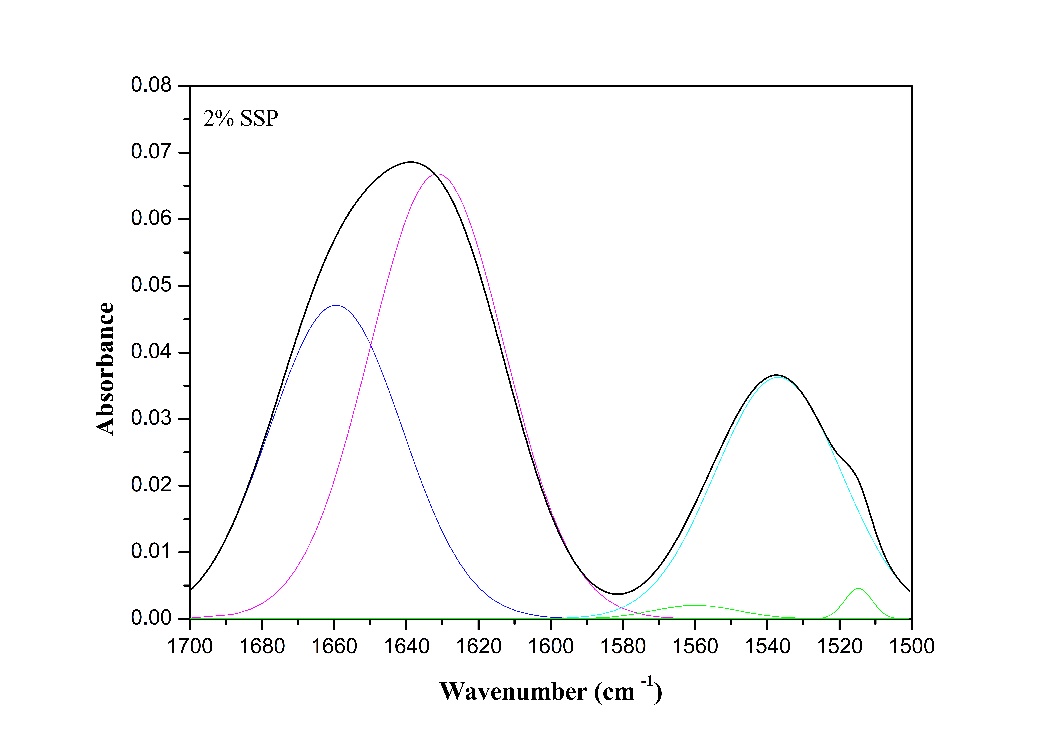


Figure S1. Curve fitting of the FTIR spectrum for the pure protein film.


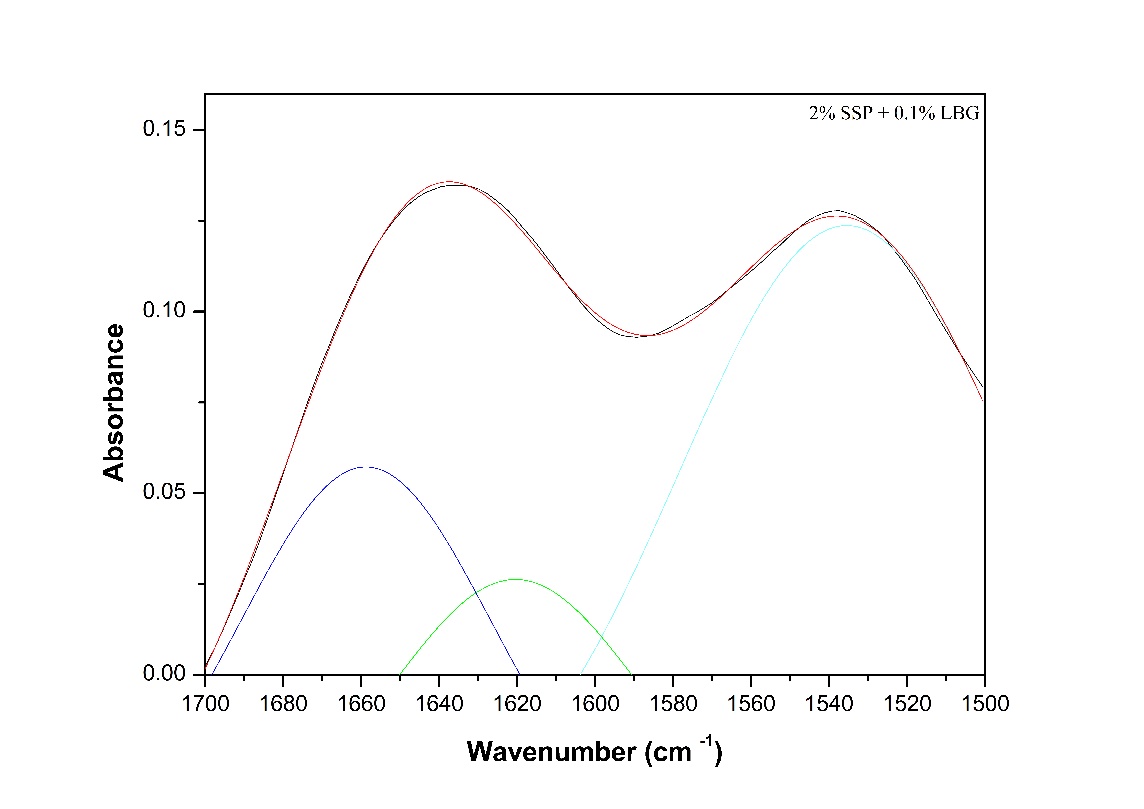


Figure S2. Curve fitting of the FTIR spectrum for the blend film with 2 % SSP and 0.1 % LBG.


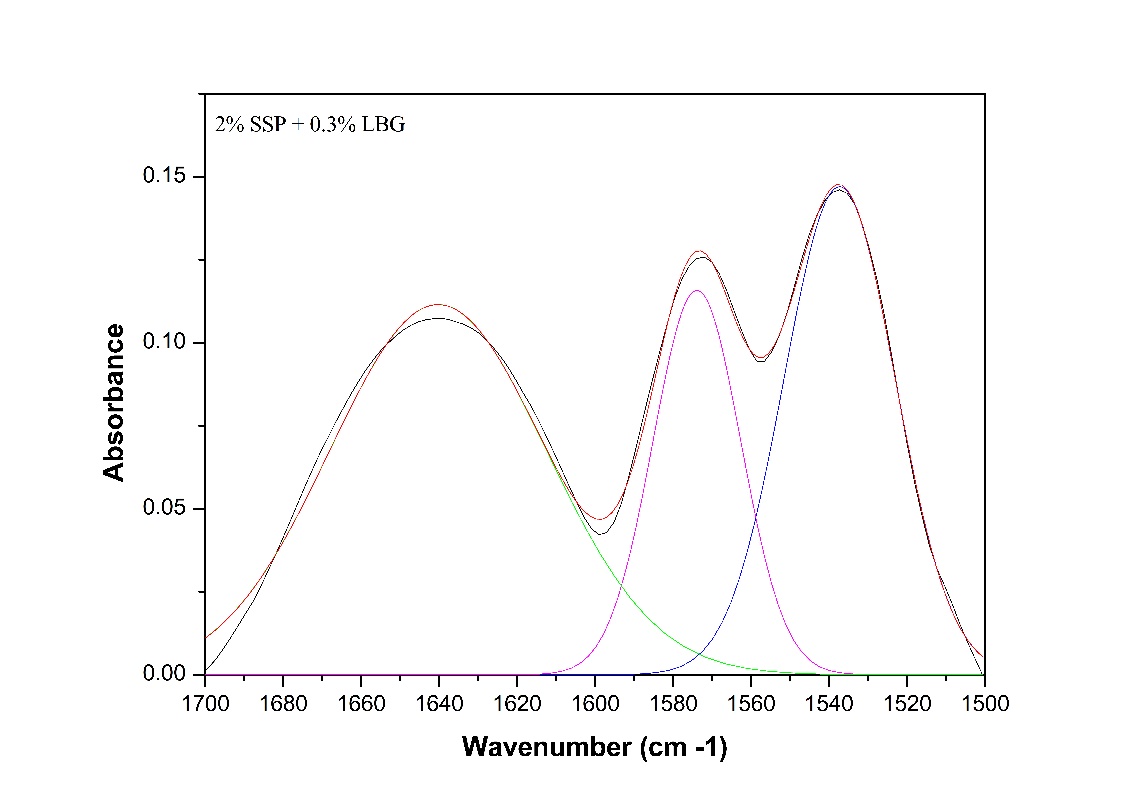


Figure S3. Curve fitting of the FTIR spectrum for the blend film with 2 % SSP and 0.3 % LBG.


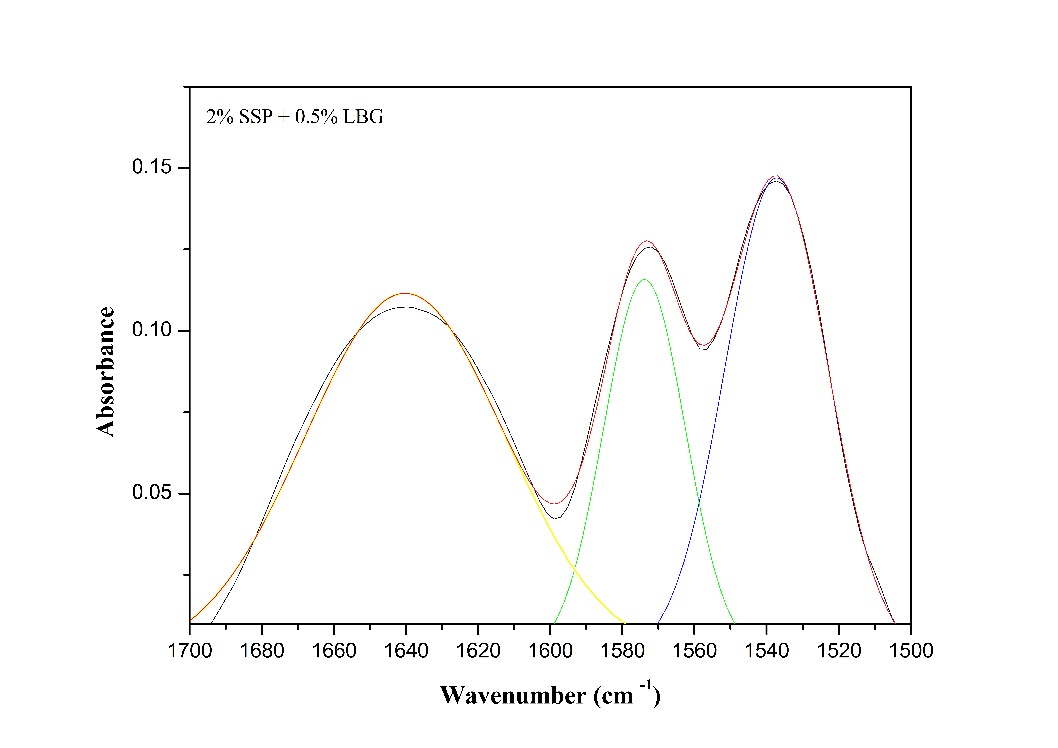


Figure S4. Curve fitting of the FTIR spectrum for the blend film with 2 % SSP and 0.5 % LBG.


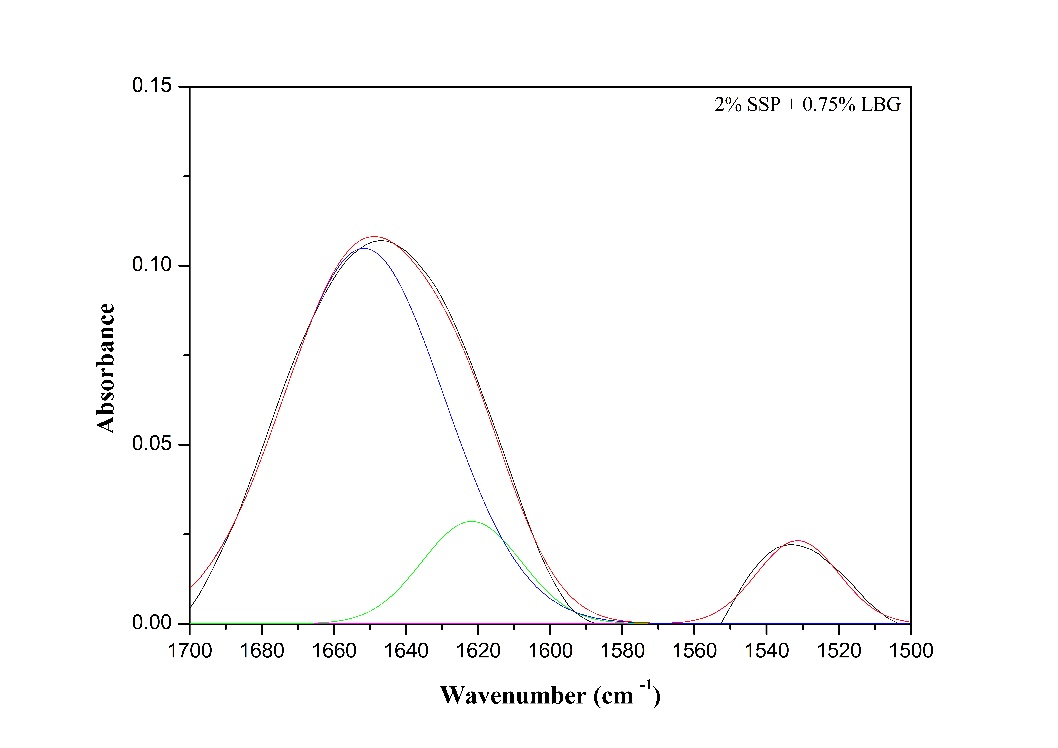


Figure S5. Curve fitting of the FTIR spectrum for the blend film with 2 % SSP and 0.75 % LBG.


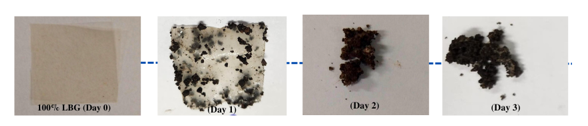


Figure S6. Biodegradability test results of film based on pure LGB.
